# Editorial: Microbial interactions of *Clostridioides difficile*

**DOI:** 10.3389/fmicb.2022.1129416

**Published:** 2023-01-06

**Authors:** Shan Goh, Peter Mullany, Thomas V. Riley

**Affiliations:** ^1^Department of Clinical, Pharmaceutical and Biological Sciences, University of Hertfordshire, Hatfield, United Kingdom; ^2^Department of Microbial Diseases, University College London (UCL) Eastman Dental Institute, Royal Free Campus, London, United Kingdom; ^3^School of Biomedical Sciences, Queen Elizabeth II Medical Centre, Nedlands, WA, Australia

**Keywords:** *Clostridioides difficile*, cell-free conditioned media, metabolites, peroxisome proliferator-activated receptor gamma, *Akkermansia muciniphila*, volatile organic compounds, SigL, IBD

*Clostridioides difficile* is a spore-forming anaerobic bacterium that causes gut infections in both human and non-human animals. In the gut, *C. difficile* colonization and pathogenicity are affected by nutrients, chemicals (e.g., metabolites), other bacteria and inflammation. Investigations of interactions with microbes and immune cells are important for effective disease treatment, and prevention and control of bacterial transmission. New strategies based on re-shaping patient gut microbiota, such as through probiotics or fecal microbiota transplantation, are effective but have biologically complex mechanisms yet to be comprehensively understood.

In this Research Topic, a collection of six articles provides a deeper understanding of *C. difficile* interactions with gut microbiota, intestinal cells and metabolites. Horvat et al. examined *C. difficile* cell-cell and supernatant-cell interactions with gut microbiota derived from healthy children through batch culture. They found both *C. difficile* cells and cell-free conditioned medium affected diversity of bacterial communities and abundance of metabolites, indicating direct and indirect ways in which *C. difficile* can influence its environment. Peroxisome proliferator-activated receptor-γ (PPAR-γ), normally expressed in adipose tissues and colonic epithelial cells, was shown for the first time to be involved in *C. difficile* infection (CDI) by Lai et al. Using mouse models, the authors showed PPAR-γ downregulation in CDI led to intestinal permeability. An agonist of PPAR-γ, pioglitazone, limited disease symptoms in *C. difficile*-infected mice. This provides a new strategy for restoring intestinal integrity during CDI. Another strategy for reshaping patient gut microbiota could be through probiotics. Wu et al. found that *Akkermansia muciniphila*, a commensal in the healthy human gut, protected against CDI in a mouse model by limiting intestinal tissue damage. *A. muciniphila* maintained microbial diversity and metabolite levels in the presence of *C. difficile* at levels similar to a healthy gut. The overall effect was colonization resistance against CDI. Currently prescribed probiotics to prevent recurrent CDI are *Saccharomyces boulardii, Lactobacillus acidophilus* and *L. casei*, however, these did not prevent primary CDI (Heil et al., 2021). Alternatives such as *A. muciniphila* could be valuable in this regard.

Metabolites of *C. difficile* are known to be important for microbial interactions, however, there have been few attempts to characterize them. Biwer et al. identified 105 volatile organic compounds (VOCs) produced as metabolites of *C. difficile* when grown in minimal medium, 28 of which were new. The authors proposed a biosynthetic pathway involving cysteine and methionine for VOC production, some of which have known effects on prokaryotic and eukaryotic cells. This study forms a basis for targeted investigations of *C. difficile* biochemical pathways corresponding to nutrient availability. In the gut, nutrients are dynamic and *C. difficile* gene transcription must be regulated in response to such changing external factors in large part through sigma factors. A previously predicted alternative sigma factor, SigL, was shown by Clark et al. to control metabolism, virulence and sporulation in *C. difficile*. Through insertional mutation of *sigL* in two isolates representing ribotypes 027 and 078, the authors revealed pleiotropic, strain-specific and growth phase-specific gene regulation in part corresponding to genetic differences of the two ribotypes. Although not examined in this study, mobile genetic elements in these strains could have contributed to strain-specific variation and would be a logical next target for further investigations.

Due to the consequences of the combination of *C. difficile* and a disrupted gut microbiome, unsurprisingly, CDI is associated with inflammatory bowel disease (IBD). Morbidity and mortality are higher in IBD patients with CDI and, with a global increasing incidence of both IBD and community-associated CDI, it is a significant problem. Mahnic et al. determined gut bacterial communities in non-IBD and IBD patients with CDI. Only in non-IBD patients, CDI was associated with lower diversity of gut bacteria compared to uninfected patients. In IBD patients, reduced gut bacterial diversity was found regardless of CDI. Interestingly, 14 differentially represented operational taxonomic units (OTUs) were common to both CDI and IBD, and four OTUs were significantly decreased in IBD patients with CDI, suggesting a role in disease severity in this patient group.

The main messages from these papers are: *C. difficile* strains can behave very differently to the same environmental stimuli, and chemicals they release have as great a role to play in modulating other bacteria and host cells as *C. difficile* toxins. Colonization is dependent on a disrupted gut microbiota and agents capable of restoring microbiome richness are effective in limiting CDI. While much *C. difficile* research has been focused on virulence and sporulation, it is equally important to study *C. difficile* in the presence of other gut constituents for a more holistic understanding of its pathogenicity.

## Author contributions

SG drafted the manuscript. TR and PM edited it. All authors approved the final version for submission.
